# Observations on the Principal Medical Institutions and Practice of France, Italy, and Germany; with Notices of the Universities, and Cases from Hospital Practice. To Which Is Added an Appendix, on Animal Magnetism and Homœopathy

**Published:** 1836-04

**Authors:** 


Art. III.— *Observations on the principal Medical Institutions and Practice of France, Italy, and Germany, with Notices of the Universities, and Cases from Hospital Practice. To which is added an Appendix, on Animal Magnetism and Homœopathy.* By Edwin Lee, Member of Roy. Coll. of Surgeons, formerly House Surgeon to St. George's Hospital. London, 1835. 8vo. pp. 216.

Although much of the information contained in this work is derived from publications easily procured in the foreign capitals, it is still such a work as must prove especially acceptable to the numerous English students resorting to the continental schools with very scanty preparation, and requiring every aid and every kind of guidance to enable them to avail themselves of the advantages there to be enjoyed, but sometimes very imperfectly known.

It is divided into three parts, devoted respectively to France, Italy, and Germany; and each part is introduced by general observations on the state of medicine in the country which forms its subject.

### FRENCH MEDICAL INSTITUTIONS.

A clear and good account of the Parisian hospitals is given (in pages 2 and 3): we think, however, that it is not correctly stated that *all* the hospitals are attended by the *sœurs de la charité* as nurses : this, however, is of small importance: the question of their efficiency is more material. Few students see the wards of foreign hospitals except during the visit of the physicians or surgeons. In the absence of these, the general service of some of the French hospitals is, we know, performed in a very slovenly manner. We have been surprised to see the house-pupils performing all the minor operations, including venesection, unattended by any nurse, even in the women's wards; and in case a patient fainted, it was sometimes necessary to summon the aid of the man who was polishing the floor by rubbing a cloth over it with his foot. Not a sœur de charité was then to be seen. Neither did it appear to us that these sisters were remarkable for the gentleness of their manners. We made these observations with regret, and in opposition to all our previous impressions; and the conclusion to which we came was, that ordinary nurses, with all their defects, were more efficient hospital attendants, such offices "pour l'amour de Dieu" being little better than certain tonsorial services recorded among authentic facetiæ as performed for a like consideration.

The following is the prescribed course of study for those who take the diploma of Doctor in medicine or surgery in Paris:

"Candidates for the diploma of Doctor in Medicine or Surgery, are required to have studied four years, during which period they have to take out an inscription every three months for attendance on the lecture and hospitals. Members of foreign colleges and universities may, however, present themselves for examination after two years' study in Paris. The scholar year begins on the 1st of November, and terminates on the 31st of August. The expense of the course of study required for taking a degree does not exceed a thousand francs (£40).

"The following is the prescribed order of study:

1st half year. Anatomy, Physiology, Chemistry.
2d ditto. Medical Physics, Hygiène, Medical Natural History.
3d ditto. Anatomy, Physiology, Operative Surgery.

4th half year. Hygiène, Medical Pathology, Pharmacy.
5th ditto. Operative Surgery, Medical and Surgical Pathology.
6th ditto. Clinical Medicine, Clinical Surgery, Materia Medica.
7th ditto. Clinical Medicine, Clinical Surgery, Medical Pathology.
8th ditto. Medical Jurisprudence, Therapeutics, Obstetricity.

"The examinations for the diploma are five in number. The first takes place after the fourth inscription has been taken out, the second after the twelfth inscription: the three remaining examinations take place at the termination of the course of study.

"The subjects of the first examination are, natural history, physics, medical chemistry, pharmacology; 2d, anatomy and physiology; 3d, general pathology, medical and surgical pathology; 4th, medical jurisprudence, hygiène, materia medica, and therapeutics; 5th, clinical medicine and surgery, operative surgery, and obstetricity.

"Each examination lasts two hours, during which four candidates are questioned by three examiners.

"For the anatomical examination the candidate is required to make a preparation from a part of the body, which is indicated to him on the same morning, and to answer questions proposed to him relative to the preparation. Candidates have also to write and defend a thesis on some point relative to medicine or surgery. The clinical examinations take place in the clinical hospital at the bedside of patients. The examination fees amount to one hundred and fifty francs." (P. 4.)

The examinations, we may add, are public, and searching and efficient, but conducted with politeness.

In France the profession is divided into physicians, surgeons, and a lower rank of practitioners called officers of health (officiers de santé), the practice of the latter being nominally restricted to cases of minor importance: we say nominally, for the restriction is plainly impracticable. Midwifery is chiefly in the hands of women, and although they are regularly educated, we think that a suspicion expressed by Mr. Lee, that this circumstance has some connexion with the great frequency of uterine diseases in France, is not quite unfounded. There are lying-in charities in our own country in which, from a spurious delicacy, women only are employed as midwives, and we have seen much of the bad effects of this regulation: it is however to be considered, that our English midwives are uneducated persons. "Apothecaries," says Mr. Lee, "are not allowed to prescribe, their business being confined to the selling of drugs, and the preparation of prescriptions, *as with chemists and druggists in England:*" who, it may be added, do not so confine themselves at all, but practise very extensively.

Mr. Lee's account of the state of medical practice in France is succinct and interesting: it is evident that French physicians are becoming less governed by theory, and more guided by symptoms; or, in other words, that the practice among them is becoming more rational. Since the publication of the opinions of M. Broussais, bleeding has been more boldly resorted to; but the French seem negligent of following up bleeding by medicines calculated to amend the secretions, or even to remove vitiated accumulations; the consequence of which is that symptoms arise which appear to call for a repetition of the bleeding, and patients are sometimes bled and starved to death. We believe this even not to be very unfrequent in fever cases, and Mr. Lee gives a striking case of typhus in illustration of it (p. 58, see also p. 26). M. Louis and M. Andral should be mentioned, however, and are so, by Mr. Lee, as exceptions to this practice; both of them employ saline purgatives and other medicines in such cases. The more frequent and varied employment of baths in chronic diseases appears to be advantageous in French practice, and is, we think, beginning to be attended to in this country. Mr. Lee very properly thinks that "the practice of abstracting blood and applying irritants at a considerable distance from the seat of the disease, on the principle of revulsion, might be more frequently adopted with advantage in England, especially in affections connected with cerebral irritation or congestion." A small bleeding from the ancle, a few leeches applied to the thighs or arms, especially to the latter, blisters to the legs, sinapisms to the feet, &c., are often singularly useful; and it is certainly to be regretted that English practitioners are often so inclined to condemn measures of this kind as trifling; we mean, of course, only in chronic cases.

In the treatment of burns and scalds, stimulating applications are less used on the continent than in England: bleeding, opium, cataplasms, or emollient dressings, being the usual means employed. Whatever is the seat of the injury, the application of ice to the head for an hour or two is strongly recommended; its effect being to cause a cessation of pain, and to prevent cerebral symptoms.

Under the head of each Parisian hospital, a concise account is given of the practice of the medical officers, and this is illustrated by occasional cases. We can only find space for a few brief extracts from this interesting part of the work.

The hospital of La Pitié has the advantage of the services of some of the greatest men in Paris, MM. Louis and Andral being the physicians; and MM. Lisfranc and Blandin the surgeons. M. Lisfranc is well known to English students for the boldness and dexterity of his practice, especially in cases of diseased uterus.

"M. Lisfranc has charge of two men's and a women's ward, most of the cases in the latter being marked as disease of the uterus; many of these patients are, however, young women affected with superficial erosion of the cervix uteri, and are cured by a few days' rest and appropriate treatment; the means resorted to in these cases, as well as in ulceration of this part, being chiefly confinement to the recumbent position, occasional venesection to three or four ounces, on the principle of revulsion, small doses of cicuta, and cauterization with a solution of mercury in nitric acid every six or eight days: when the ulceration is of a cancerous nature and too deep to be removed by cauterization, M. Lisfranc has recourse to excision of the cervix uteri; this part being exposed by the speculum, and firmly seized by *pinces de museaux*, is brought down beyond the orifice of the vagina and excised with a knife, as in cases of polypus. M. Lisfranc has not met with more than four or five cases of dangerous hæmorrhage after this operation, the symptoms which supervene being mostly of a nervous character and sometimes alarming, but mostly yield (yielding) to sedatives. Of ninety-nine cases in which he operated, eighty-four recovered: many of these patients became subsequently pregnant, and experienced no particular inconvenience in parturition." (P. 64.)

Mr. Lee adds, what few will doubt, that the operation has been performed in many cases in which it might have been avoided. M. Lisfranc treats *phlebitis* (supervening on venesection) by emollient fomentations and cataplasms on the inflamed part, with the repeated application of leeches between the point where the inflammation terminates and the heart; and he states, that since he adopted this practice, he has not lost a single patient from this disease; whereas when he employed other means, and applied leeches near the wound or over the inflamed vein, the majority of cases terminate unfavorably.

MM. Alibert and Biett, the physicians to the hospital of St. Louis, are also well known to English practitioners; the first by a splendid publication on diseases of the skin, the second by his enlightened practice in cutaneous, scrofulous, and malignant affections. The opinions of M. Biett on the use of baths in cutaneous diseases may be useful to the English reader.

"Simple tepid baths are most beneficial in the dry scaly forms, though only as an accessory means; their efficacy is less marked in the pustular varieties: they are serviceable in vesicular affections when the inflammation begins to decrease, and may be used with advantage in impetiginous affections where incrustations have succeeded to the pustules.

"Alkaline baths are efficacious in the papular and dry scaly forms, and in the impetiginous and tubercular varities. An alkaline bath may be formed by dissolving in a simple bath from half a pound to a pound of carbonate of soda.

"Sulphur baths are most useful in the decline of vesicular affections: they are less useful than alkaline baths in the chronic stage of psora, and if used in the inflammatory stage the symptoms are aggravated. Sulphurous baths are composed of two ounces of diluted sulphuric acid and eight ounces of hydro-sulphuret of potass added to each bath.

"Acid baths may be made by adding to each from four to eight ounces of hydrochloric acid; they are mostly applicable in dry scaly eruptions." (P. 69.)

In the account of the Hôpital des Enfans Malades (p. 75), we perceive that the mortality among the unfortunate children is stated to be one in four, which we believe to be rather under than over the truth. According to our own painful observation, this horrible mortality is to be ascribed, in a great measure, to the *expectant* system of medicine; to starvation, and gum-water, conjoined with the too free use of leeches.

M. Civiale, whose name is familiar to us in connexion with lithotrity, has a small ward in the Necker hospital, and the following is mentioned in the account of his practice.

"Paralysis of the bladder, and vesical catarrh, in elderly people, are treated in the following manner:—a stream of cold water flows from a reservoir fixed near the ceiling, through an elastic gum tube, having stop-cocks, and terminating in a silver catheter formed into a double tube by a central partition. The patient being in the recumbent position and the catheter introduced, the water passes into the bladder by one side and out by the other. A continued stream of water through the bladder is thus kept up for about ten minutes, and repeated every second or third day: the quantity of water passing into the bladder may be regulated by the stop-cock, so as to prevent undue distention. The beneficial effects of the method are attributed to the clearing away of the accumulated mucus, and to the tonic action of the cold water upon the bladder." (P. 79.)

### ITALIAN MEDICAL INSTITUTIONS.

Mr. Lee's observations under this head are somewhat brief. The different states and cities of Italy, as remarked in our notice of the Foreign Reviews and Journals, in our first Number, are like so many different countries; and we accordingly find that the general state of medical, as of all other knowledge, is unequal. At Florence and Rome the Broussaian doctrine prevails; but it is in less repute at Milan and at Naples. The Rasorian doctrine, and the practice of administering large doses of antimony instead of bleeding, are on the decline. Mr. Lee tells us that not only lithotrity, but auscultation and percussion meet with no advocates among the Italians, who seem averse to every innovation. In some parts of Italy, surgeons are only permitted to apply local remedies, and perform operations, physicians being called in to prescribe for the constitution, even in surgical cases. Perhaps it is the consequence of this division of labour, that the surgeons generally manage their cases without any medicine, by which plan many patients sink under constitutional irritation, or internal inflammations. These circumstances do not prepare us to find much that is interesting, under the head of the different hospitals. In some of them, indeed, Mr. Lee seems to have made few personal observations: and neither Foreign nor English readers can be much satisfied with such a remark, applied to the principal hospital at Geneva, as, "I did not see much of the practice, but believe it to be very inferior."

Several interesting statements are given, relative to the management of the insane in the different institutions. In some of them, as in the Hospital of Incurables at Geneva, it is still exceedingly defective. About 250 patients are confined to ill-ventilated wards, which they never leave until they die, or are dismissed. There is no classification of the cases, and except occasional depletion, curative measures seem little attended to. Mr. Lee was not allowed to see the women's wards; the probable condition of which, therefore, is not better than when described by M. Brière de Beaumont, at which time the women were shut up in dirty and ill-lighted wards, one of which was large, cold, and damp; and many of the patients were chained by the hands and feet, so that "their howlings, their accessions of fury, and the clanking of their chains, gave to this horrible place the appearance of the infernal regions." From the end of 1828 to the end of 1829,

| | Men. | Women. |
|---|---|---|
| The admissions were . . | 90 | 84 |
| There were dismissed . . . | 40 | 32 |
| Died . . . | 32 | 32 |

The proportion of deaths to the number of inmates (about 250,) is, Mr. Lee remarks, enormous. (P. 93.) The treatment of this unfortunate class of patients can hardly be said to be in any degree better in the Hospital for the Insane, at Rome. (P. 123.) At Bologna it is much better; (P. 100,) and also at the Spedale di Bonifacio, at Florence, (P. 105,) where each patient has a separate cell, and is allowed to walk about in the day time: the new patients are kept in separate rooms for a few days, that the peculiarities of their insanity may be observed; and the greatest attention is paid to cleanliness throughout the establishment. Furious patients are consigned to darkened rooms, with well-padded walls; and the darkness is said to have the effect of making a great number of them tractable. Many of the patients are employed in mechanical operations, gardening, knitting, spinning, &c.; and in this hospital the ancient custom of bleeding all the patients in summer, is discontinued. The superiority of the establishment for the insane at Aversa, near Naples, seems to have been exaggerated; but some of the arrangements, and the general order and regularity of the institution, are excellent.

The school of Bologna boasts of having produced Valsalva, Malpighi, and Galvani: the population of the place is, even now, only 75,000; so that we may not unreasonably expect similar advantages from some of our own provincial schools, for the production of which it is evident that the air of a large capital is not indispensable. The order of study, and the mode of examination, are worthy of quotation.

"Medical students are obliged to attend the classes during four years, in the following order:—first year, Natural History, Botany, Chemistry, Anatomy; second year, Anatomy, Physiology, Comparative Anatomy, Institutes of Surgery; third year, Pathology, Clinical Medicine, Materia Medica, Chemistry; fourth year, Pathology, Clinical Medicine, Medical Jurisprudence, and Midwifery. During the last year of study, a certain number of patients are placed under the care of each pupil, who, previous to his examination, has to give an account of the cases, and of the treatment he has adopted. Surgical students attend, during the first and second years, the same courses of lectures as the medical pupils; third year, Institutes of Surgery, Clinical Surgery, Anatomy, and Dissections; fourth year, Medical Jurisprudence, Midwifery, Dissections, Clinical Surgery, and the performance of operations on patients, under the guidance of the professor. At the termination of the first year, students take the degree of bachelor; at the end of the second year, of licentiate; and at the end of the fourth year, of doctor of medicine and surgery.

"The mode of examination of candidates is as follows:—five professors of the different branches of education submit each to the candidate twenty different subjects, taken from his own course of instruction; the pupil draws one of these by lot, and is examined on that subject. Thus the candidate is examined on five subjects connected with medicine. When the examination is finished, each of the professors gives his vote separately, as to the fitness of the candidates; those who are considered not sufficiently qualified, have to study another year." (P. 102.)

### GERMAN MEDICAL INSTITUTIONS.

As we are enabled to refer to the Fourth Part of this Journal, for authentic details respecting the state of medicine and medical institutions in Germany, we shall pass over many interesting observations in this part of Mr. Lee's work. The general system of medical practice approaches much more nearly to that of England than in France and Italy; bleeding is less frequently had recourse to, but active internal medicines are employed, as well as enemata and baths, whilst tisanes, and infusions of herbs, are now but little prescribed.

When speaking of the hospital at Stuttgard, Mr. Lee mentions that sciatica is treated, in that institution, not by blisters applied over the trunk of the sciatic nerve, but below the knee, so as to encircle the leg; a method which is said to produce a speedy cure in most cases. In the general hospital at Prague, rheumatism is treated by the administration of large quantities of warm water: both acute and chronic cases appear to be treated in this manner, and it is said with success. In a work by Dr. Brandis, published at Berlin in 1833, that physician recommends cold bathing, and even cold affusions, in rheumatism: a curious instance of the uncertain state of physic!

One of the best establishments on the Continent, for the reception of insane patients, is that of Sonnenstein, about four leagues from Dresden, of which a very good account is given by Mr. Lee, (p. 151.) It consists of an ancient castle, seated on a hill, and having extensive grounds. The number of patients, at the time of Mr. Lee's visit, was 200, of whom 120 were men. The house is extremely clean, and contains workshops, a saloon of amusement, with books, a piano, draught-boards, and a billiard room. In fine weather the men are employed out of doors, and the women amuse themselves with a flower-garden. The furious patients are not, as in the Italian institutions, confined to dark rooms, "but are allowed to walk about with the rest, their hands being confined. They also wear a cloak, in order that the apparatus for confining the hands may not be observed by others. This method is found to have greater effect in tranquillizing them, than if isolated, and forcibly confined to bed." (P. 153.)

The cures at Sonnenstein are reported to be one in three among women, and one in four among the men. Paralysis is observed less frequently to supervene on insanity than in France.

As a melancholy contrast to Sonnenstein, we may take the insane wards in the Kranken-Haus, at Berlin; where 150 patients are kept entirely in the house, having no ground for exercise; and where the general treatment appears to be quite neglected. Yet the other departments of this hospital are well regulated. The treatment in the syphilitic wards consists, chiefly, in rigid abstinence; bread, soup, and milk only being allowed, and even of these only a quarter of the ordinary allowance of other patients. Mercury is never employed: the usual medicines given are the neutral salts, especially the sulphate of magnesia, and sarsaparilla. The applications to sores are emollient, slightly stimulating, or caustic; and the usual duration of the treatment is three or four weeks. Secondary symptoms seldom occur, and are not of a serious nature.

Professor Langenbeck, "one of the most distinguished anatomists and surgeons of Germany," resides at Gottingen, the university of which little town was founded in 1734, by George the Second. At this famous university, in a town containing only about 10,000 inhabitants, there are no fewer than 1,500 students, and *eighty* professors. Until lately, the celebrated Blumenbach, who is still living, was professor of physiology; and his rich collection of crania, and fossil remains, including precious specimens from the Hartz mountains, give value to the museum. Gottingen has also the great advantage of possessing a library of great extent, admirably arranged; a celebrated observatory; and one of the best botanical gardens in Germany. When will any of our moderate sized English country-towns possess such advantages?

Mr. Lee has appended to his work some account of animal magnetism and homœopathy, on which we do not think it necessary, on the present occasion, to make any comments; our principal object in this notice having been to shew the student the kind of information contained in Mr. Lee's book. Altogether, it is certainly a publication which we would recommend every student about to proceed to the Continent to put into his portmanteau. There is still, we think, a book wanting for students, containing a well arranged view of the advantages peculiar to each of the continental schools; the time of the year when the lectures are given; the best mode of living to be adopted in the several towns, and the expenses likely to be incurred; with particular references to the character of the different anatomical collections, or parts of such collections. Even a medical map would be a useful accompaniment to such a work. A plan should also be given for six months', or a year's, or two years' travel and medical study on the Continent. Such a book, however, could not be written by a mere tourist, or without devoting a longer period to observation of the state of the different continental schools than we could expect any single observer to have time and opportunities for. But materials for it might be contributed by different observers, each taking for the subject of his remarks, the country or the school with which he possessed the means of becoming most intimately acquainted. We may add, that the Fourth department of our Journal will always be open to contributions conveying this very useful kind of intelligence.

Even more generally useful, perhaps, to English students, would be a work containing clear directions how to pass the months of a single winter profitably, in the Parisian schools. It often happens that our young countrymen, on arriving in Paris, being imperfectly acquainted with the French language, and unprovided with useful introductions, lose some time in making preliminary arrangements, and make them injudiciously. The distance of the hospitals from each other, or their relative position; their respective merits; the choice of a residence during attendance upon some of them part of the time, and then upon others; are all points of great consequence to a student whose time and whose resources happen to be limited. In every student's life, too, however busy, there are unemployed hours, and in no city may these be more advantageously passed than in Paris, either in the public resorts of men of science, or in improving private society, to which respectability of character, and a love of knowledge, furnish there a sufficient introduction. Pursuing the same idea, we should be glad to see directions for passing nine months in Germany, and three months in Italy. Above all things, the English student should prepare himself, by a competent knowledge of the language of the country he means to visit, without which he cannot but be exposed to continual mortifications, and serious disappointment.

